# Efficacy and safety of dietary supplements for the treatment of ulcerative colitis, a network meta-analysis

**DOI:** 10.3389/fmed.2026.1816535

**Published:** 2026-04-22

**Authors:** Rong Xu, Zhiyu Zhang, Ying Li, Zhijiu Wu

**Affiliations:** 1 Affiliated Hospital of North Sichuan Medical College, Nanchong, China; 2 North Sichuan Medical College, Nanchong, China

**Keywords:** complementary medicine, dietary supplements, network meta-analysis, randomized controlled trial, ulcerative colitis

## Abstract

**Background:**

In recent years, the application of dietary supplements in the treatment of ulcerative colitis (UC) has attracted more and more scholars’ attention, and a number of studies have shown that dietary supplements have a non-negligible role in improving the clinical symptoms and quality of life of UC patients. This article aims to compare the efficacy and safety of dietary supplement therapy of UC through a comprehensive network meta-analysis method.

**Methodology:**

A comprehensive literature search was conducted in multiple databases, including PubMed, the Cochrane Library, Embase, and Web of science, from their inception up to 30 October 2025. Network and routine meta-analyses using the mvmeta package in Stata 16.0 and Stata 12.0 software, and literature quality and risk of bias assessed using RevMan 5.3 software.

**Result:**

A total of 24 randomized controlled trials were conducted with 1,491 participants and 14 dietary supplements. The results showed that probiotics [MD = −1.64, 95% CI (−2.91 to −0.38)] and selenium [MD = −2.43, 95% CI (−4.85 to −0.01)] had significant advantages compared with conventional medical treatment or placebo in reducing clinical activity index. Flaxseed extract [MD = −1.53, 95% CI (−2.82 to −0.24)] and vitamin A [MD = −1.93, 95% CI (−3.82 to −0.04)] had a clear advantage over conventional medical treatment or placebo in reducing the Mayo score; at the same time, in terms of IBDQ, probiotics [MD = 7.78, 95% CI (1.39 to 14.17)], flaxseed extract [MD = 8.58, 95% CI (2.31 to 14.85)], resveratrol [MD = 9.18, 95% CI (2.45 to 15.91)], curcumin [MD = 25.80, 95% CI (2.00 to 49.60)], butyrate [MD = 20.50, 95% CI (9.80, 31.20)] with higher scores compared to conventional drugs or placebo, indicating an improved quality of life for patients; synbiotics [MD = −0.69, 95% CI (−1.16 to −0.23)], flaxseed extract [MD = −0.8, 95% CI (−1.16 to −0.44)], and *Achillea wilhelmsii* [MD = −2.95, 95% CI (−3.52 to −2.38)] all showed significant advantages compared with conventional medical therapy or placebo for reducing C-reactive protein levels.

**Conclusion:**

Various dietary supplements have shown differentiated efficacy in adjuvant UC therapy. Probiotics and selenium are effective in improving clinical symptoms; flaxseed extract showed consistent benefits in improving Mayo score, IBDQ score, and reducing CRP, ESR, and FCAL, and had a wide range of application prospects. Butyrate and curcumin have significant effects in improving quality of life; synbiotics and *Achillea wilhelmsii* have outstanding advantages in reducing CRP. Due to the limitations of the number and quality of included studies, the above conclusions still need to be validated by more high-quality, large-sample RCTs.

## Introduction

Ulcerative colitis (UC) is a chronic inflammatory bowel disease that usually presents with successively inflamed colonic segments involving the distal rectum and extending proximally (1). Its main clinical manifestations are persistent or recurrent diarrhea, mucus, pus and bloody stools with abdominal pain, tenesmus and heaviness, which may be accompanied by fatigue, loss of appetite, fever and other systemic symptoms. Epidemiological characteristics show that the incidence of UC is on the rise worldwide, especially in high-incidence regions such as North America and Europe (2). In addition, patients with UC may have an increased risk of colorectal cancer due to chronic inflammation, and long-term changes in serum inflammatory marker levels are closely related to the occurrence of colorectal cancer (3). Studies have shown that the wider the range of inflamed colon segments, the higher the risk of colorectal cancer, especially in those with generalized colitis and left-sided colitis (4). At present, the first line of drug regimens includes 5-aminosalicylic acid (5-ASA), glucocorticoids, immunomodulators and biologics, etc., which have achieved certain efficacy in inducing and maintaining clinical remission of UC and promoting mucosal healing, but at present, these drugs are prone to hormone dependence and hormone resistance, gastrointestinal reactions, headache, rash, fever, hypersensitivity, hepatotoxicity, opportunistic infections and hematological diseases and other adverse reactions, and the incidence is high (5). Although surgical colectomy is considered curative, ulcerative colitis currently lacks a definitive medical cure through pharmacological interventions alone. Consequently, how to effectively alleviate UC symptoms, delay disease progression, reduce recurrence rate and improve patients’ quality of life has become an urgent clinical problem to be solved (6, 7).

In recent years, the application of dietary supplements in the treatment of UC has attracted more and more attention from scholars, and a number of studies have shown that dietary supplements have a non-negligible role in improving the clinical symptoms and quality of life of UC patients, such as vitamin D, fish oil, probiotics, prebiotics, curcumin, etc., which are considered to have certain efficacy and safety in the treatment of UC (8, 9). These dietary supplements may have a positive impact on the disease control of UC patients by modulating the gut microbiota, reducing inflammatory responses, and other mechanisms. The study (10) showed that vitamin D can significantly improve the clinical efficacy of UC patients by improving gut barrier function and reducing inflammatory factor levels. The combination of probiotics and prebiotics (i.e., synbiotics) is thought to significantly improve clinical indicators in UC patients, and synbiotics can not only improve colonoscopy and histology scores, but also reduce the level of inflammatory markers and improve the intestinal flora structure of patients (11). In addition, curcumin, as a natural polyphenolic compound, has also received widespread attention for its adjuvant therapeutic effect in UC, and studies have shown that curcumin can effectively induce clinical remission in UC patients, and its safety is good at the appropriate dose and mode of administration (12, 13). However, although dietary supplements have shown some potential in the treatment of UC, their efficacy and safety still need to be verified by more high-quality randomized controlled trials. As a method of comprehensively analyzing the results of multiple studies, network meta-analysis can provide more comprehensive evidence support for the application of dietary supplements in the treatment of UC (14, 15). Based on the above studies, we believe that dietary supplements can be used as an important treatment method to improve UC patients, which can effectively cooperate with the use of clinical first-line drugs and effectively reduce inflammation and clinical symptoms in UC patients.

## Search strategy

We conducted a comprehensive literature search in multiple databases, including PubMed, Cochrane Library, Embase, and Web of science, and searched relevant reviews and other meta-analyses to complement existing research, up to 30 October 2025. The literature screening and data extraction process was carried out independently by two reviewers (RX and ZZ), and any disagreements were resolved through consultation with the third reviewer (YL). We referred to previous meta-analyses of the same kind to refine our search strategy. First, the retrieved article titles and abstracts were screened to exclude irrelevant studies. Subsequently, the full text of the remaining articles was evaluated to determine if they met the requirements based on the inclusion criteria. A complete search strategy is detailed in the Supplementary material. This strategy was originally developed for the PubMed database and was subsequently adapted to fit the specific characteristics of other databases. We designed and wrote this paper according to the Preferred Reporting Items for Systematic Reviews and Meta-Analyses (PRISMA) 2020 statement (16), and registered the protocol with PROSPERO (CRD420261323910).

## Materials and methods

### Inclusion and exclusion criteria

Our inclusion and exclusion criteria were established in accordance with the PICOS framework. The PICOS principle is presented in Table 1. The inclusion criteria for the following studies are as follows: (1) Meet the diagnostic criteria for UC (1); (2) Age ≥18 years; (3) The treatment group must include a dietary supplement, while the control group should receive conventional UC therapy, a placebo, or lifestyle modification alone; (4) Outcome measures must include pre- and post-treatment assessments of the UC Clinical Activity Index, Mayo score, Inflammatory Bowel Disease Questionnaire (IBDQ) score, fecal calprotectin (FCAL), C-reactive protein (CRP), and erythrocyte sedimentation rate (ESR); (5) Study design: randomized controlled trial (RCT).

**Table 1:** PICOS criteria.

Criteria	Explanation
Population	Adults (≥18 years) with UC
Intervention	Specific dietary supplements (curcumin, probiotic, etc.)
Comparator	Conventional therapy, placebo, or no intervention
Outcomes	Clinical disease activity, inflammatory bowel disease quality of life score, Mayo score, FCAL, CRP, ESR
Study design	RCTs

The following criteria will be used to exclude studies from consideration: (1) Duplicate publications or studies with overlapping data; (2) Non-human studies (e.g., animal or *in vitro* studies); (3) Studies involving pregnant women or children; (4) Publications without sufficient data for analysis or without full-text availability (e.g., conference abstracts, letters, case reports); (5) Not RCT.

### Data extraction and quality assessment

Two reviewers (RX and ZZ) independently performed the literature search, study selection, and data extraction using a standardized data collection form. The extracted data included: author names, publication year, sample size, participant age and sex, intervention details, treatment duration, and outcome measures. Any discrepancies were resolved through discussion with a third reviewer (YL) to reach a consensus. In cases where study data were incomplete or missing, the corresponding authors were contacted to obtain the necessary information. Studies were excluded if the required data could not be obtained. The risk of bias in RCTs was assessed using the RCT bias risk assessment tool recommended in the Cochrane Handbook version 5.1.0. The following domains were evaluated: (1) Random sequence generation (selection bias); (2) Allocation concealment (selection bias); (3) Blinding of participants and personnel (performance bias); (4) Blinding of outcome assessment (detection bias); (5) Incomplete outcome data (attrition bias); (6) Selective reporting (reporting bias); (7) Other sources of bias. Each domain was rated as “low risk,” “high risk,” or “unclear risk” of bias based on the criteria outlined in the Cochrane Handbook.

### Statistical analysis

This study employed the mvmeta package in Stata 16.0 and Stata 12.0 software for network and routine meta-analyses, using mean ± standard deviation as effect size metrics for quantitative data analysis. Literature quality and bias risk were evaluated via RevMan 5.3 software, all effect indicators were presented with 95% confidence intervals (CI). Heterogeneity across studies was assessed by visually inspecting the prediction interval plot. Local inconsistency was evaluated using the node-splitting method. The similarity assumption of the evidence network was examined by comparing the clinical and methodological characteristics of the included studies. A league table was constructed to present the comparative effects of all pairwise interventions for each outcome. The statistical significance level for all meta-analyses was set at *α* = 0.05. For closed loops formed by multiple interventions, inconsistency testing was performed to assess the degree of agreement between direct and indirect evidence. The relative ranking of interventions was estimated using the surface under the cumulative ranking curve (SUCRA) probabilities, allowing identification of the most effective intervention. The SUCRA is used to evaluate the probability of the relative efficacy ranking of different interventions based on the current evidence network, reflecting only “relative possibility” rather than “absolute clinical advantage.” This indicator is an exploratory analysis tool, and its results need to be combined with the statistical significance of pairwise comparison, research quality, sample size, publication bias and other comprehensive judgments. It should not be used as an authoritative basis for clinical optimal intervention selection alone. Publication bias was evaluated by visual inspection of a comparison-adjusted funnel plot, generated using Stata version 16.0. For multi-arm trials, data processing and analysis were performed by merging subgroups following the approach used for two-arm trials, or by stratifying according to different intervention arms as appropriate. To address potential inconsistency arising from variations in control groups across the included studies, a sensitivity analysis was conducted. Specifically, we performed an additional analysis by excluding trials that did not use a strict placebo control, such as studies utilizing active drugs or undefined conventional therapies as comparators.

## Results

### Literature search details

**Figure 1:**
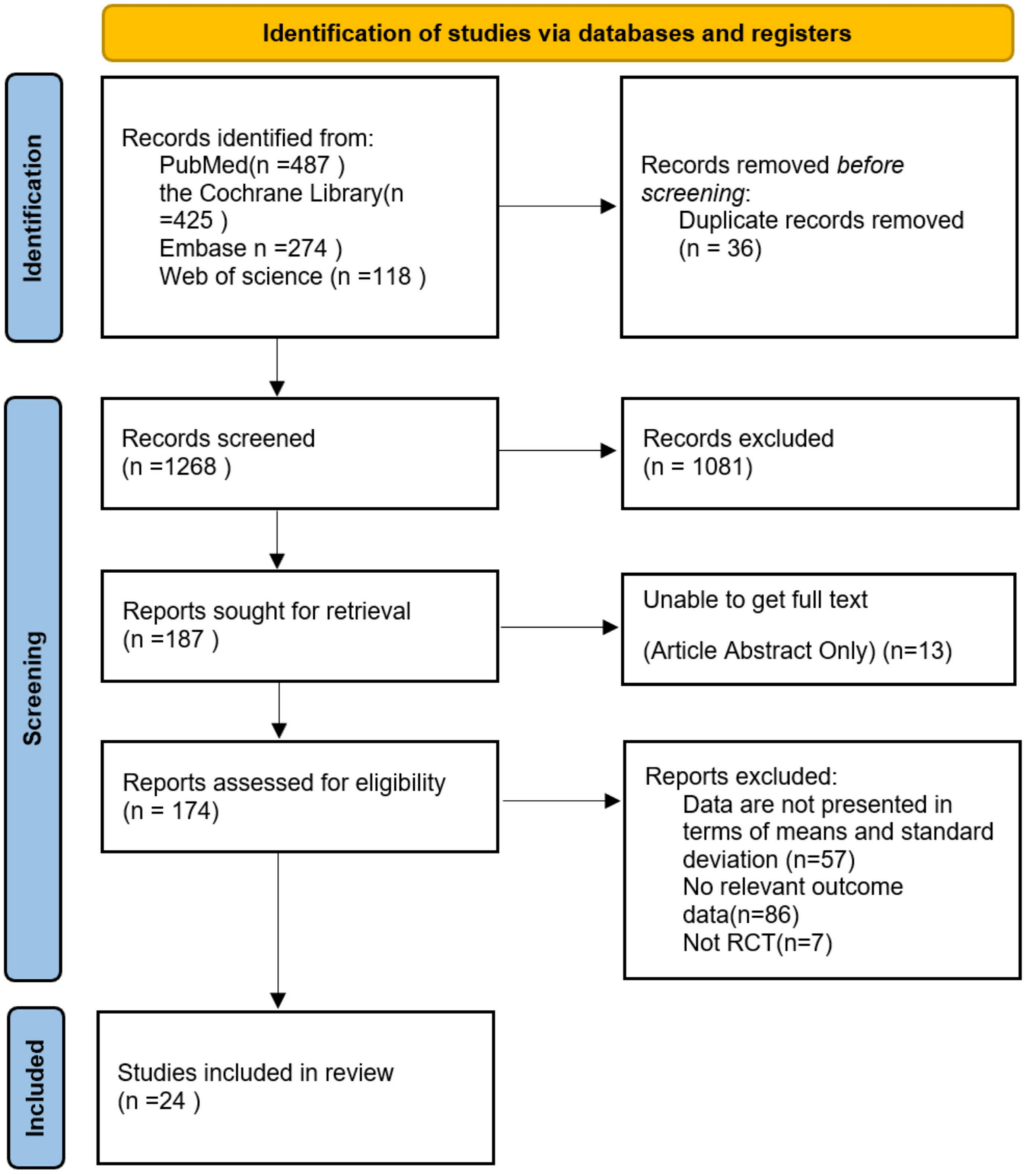
Document screening process and results.

Figure 1 illustrates the literature screening process. The search process and study selection were documented using a PRISMA flowchart. Initially, 1,304 records were identified. After title and abstract screening, 174 full-text articles were assessed for eligibility. Of these, 150 were excluded for various reasons, and the remaining 24 studies satisfied the inclusion criteria and were included in the meta-analysis, collectively involving 1,491 participants. The publication years of these studies range from 2004 to 2025.

### Study characteristics classification

We incorporated a total of 24 (17–40) studies and all studies are RCTs. These studies involved conventional treatments and 14 dietary supplements, including probiotic, prebiotics, synbiotics, *Coptis chinensis*, resveratrol, curcumin, flaxseed extract, butyrate, spirulina, *Achillea wilhelmsii*, vitamin B6, selenium, vitamin A, ginger extract. The fundamental characteristics incorporated in the study are presented in Supplementary Table S1.

### Literature quality evaluation

**Figure 2:**
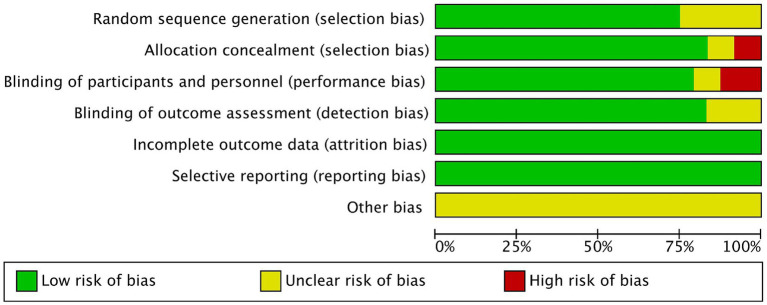
Percent of studies with categorize for risk of bias.

**Figure 3:**
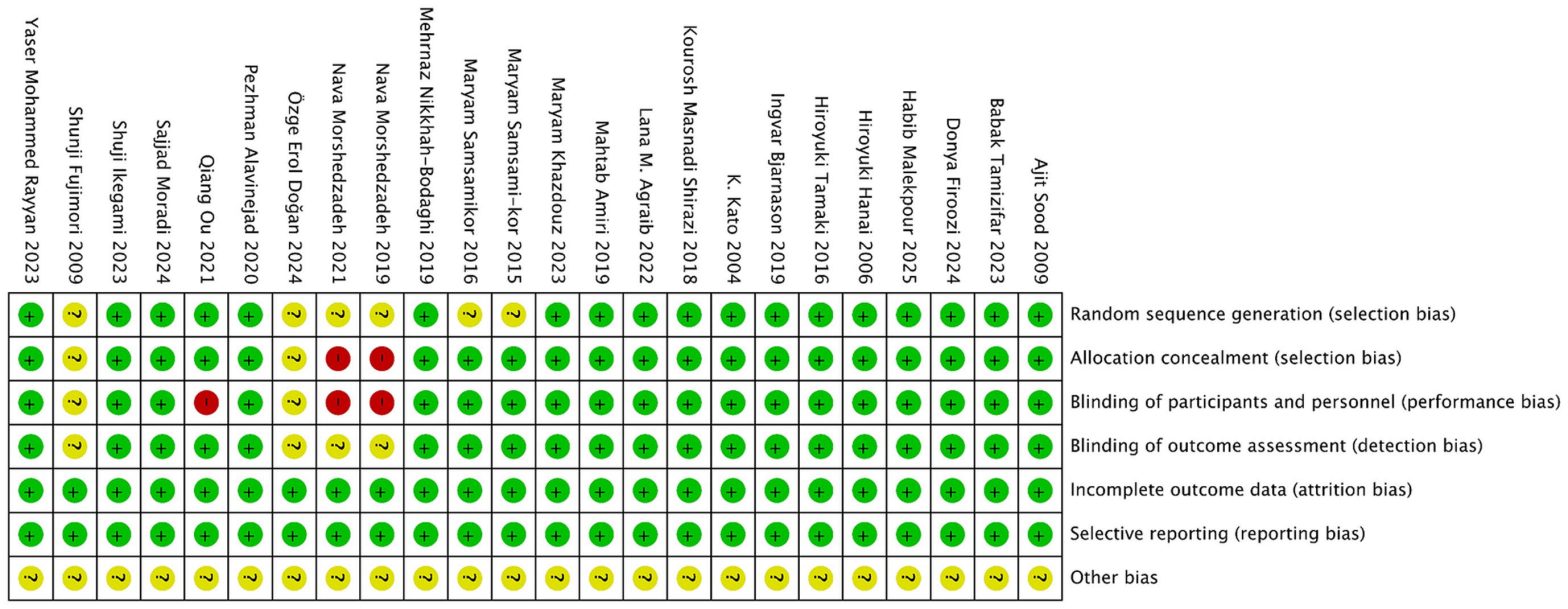
Summary of the risk of bias in each study.

We performed a quality assessment of the 24 RCTs included in our meta analysis. An independent assessment was conducted by two authors to ensure the credibility of the evaluation. In instances of disagreement, a third author made the final decision. Regarding random allocation methods, 18 studies were classified as low-risk due to their use of random number tables or computer generated random allocation sequences (17, 19, 21–23, 26–33, 35, 37–40). Conversely, six studies merely mentioned randomness without specifying the allocation scheme, resulting in an unclear risk classification (18, 20, 24, 25, 34, 36). We found that 19 studies provided detailed reports on allocation concealment and blinding procedures, including the use of double-blind methods, and were thus rated as low-risk (17, 19–22, 26–35, 37–40), in contrast, one study reported only subject blinding (23), and two studies opted for an open-label design and were rated as high-risk (24, 36). Two studies did not address allocation concealment and blinding, leading to an unclear risk designation (18, 25). All studies presented complete research data. To assess selective reporting, we verified the consistency between the methodology and results sections of each study, finding all to be low-risk and fully reported. However, other potential sources of bias remain unclear. The bias risk assessment was conducted using RevMan 5.3 software, as illustrated in Figure 2, with detailed results available in Figure 3.

### Network meta-analysis

#### Clinical activity index

**Figure 4:**
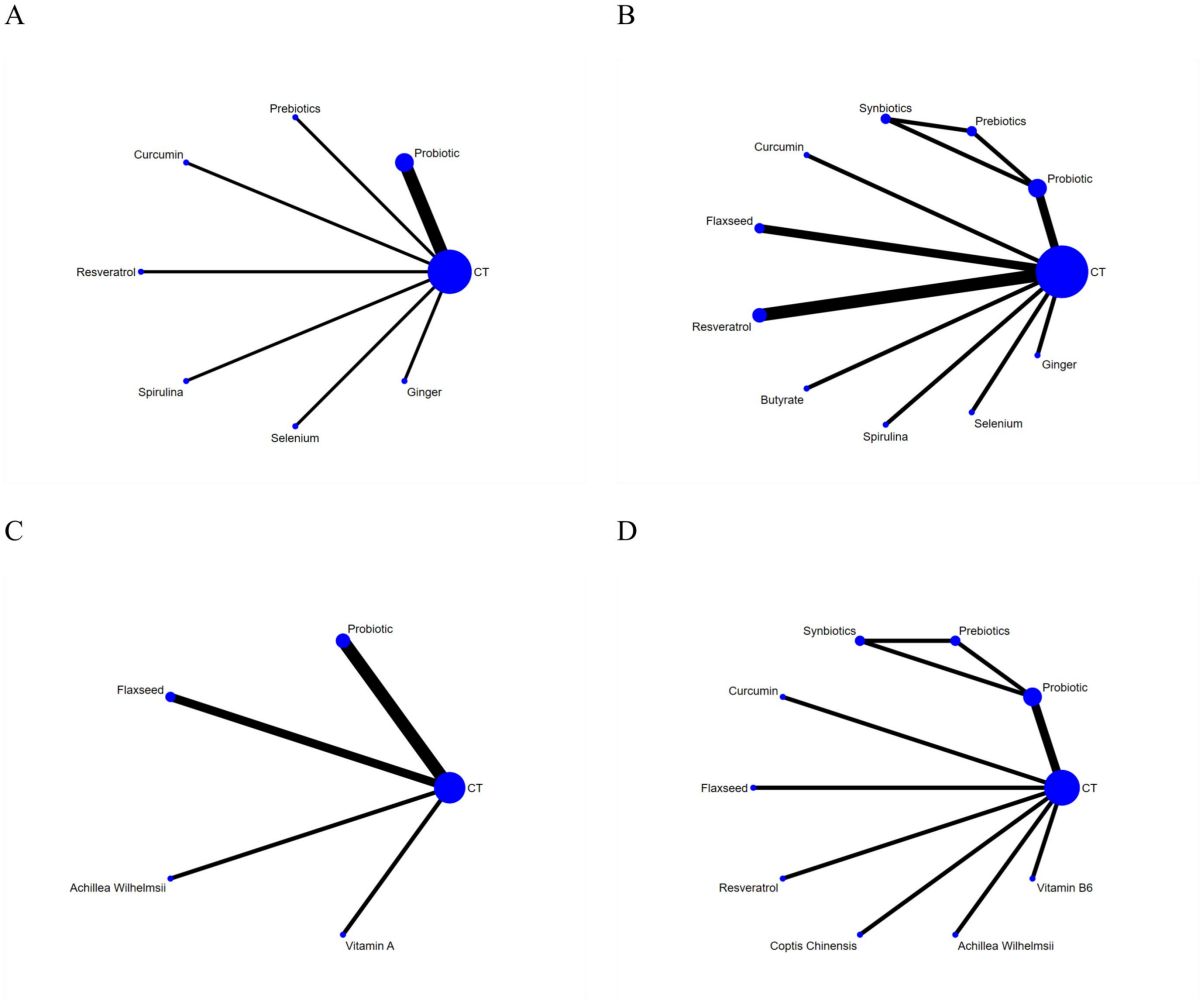
The evidence network of all papers on different outcomes. **(A)** Clinical disease activity. **(B)** IBDQ score. **(C)** Mayo score. **(D)** CRP. (a) The abbreviation CT represents conventional treatment or placebo. (b) The size of the nodes is proportional to the number of randomized participants, and the thickness of the edges is proportional to the number of included studies evaluating that specific comparison.

A total of nine studies (17, 20, 21, 27, 29, 33, 35, 38, 40) reported on the improvement of clinical activity index in patients with UC with a total of 567 patients involving seven interventions. The network relationships among these interventions are depicted in Figure 4A. If the inconsistency model test yields a *p* > 0.05, the consistency model is deemed appropriate for NMA. The NMA revealed that, in terms of clinical activity index, among seven dietary supplements compared with conventional drug therapy or placebo, probiotics (MD = −1.64, 95% CI: −2.91 to −0.38) demonstrated significant superiority, with statistically significant differences (*p* < 0.05). Selenium (MD = −2.43, 95% CI: −4.85 to −0.01) also showed a statistically significant reduction in CAI; however, because the upper bound of the confidence interval barely excludes zero, this potential benefit is marginal and should be interpreted with caution. No statistically significant differences were observed in other pairwise comparisons among the remaining interventions (*p* > 0.05). Detailed results of the pairwise comparisons are provided in Supplementary Table S2.

**Figure 5:**
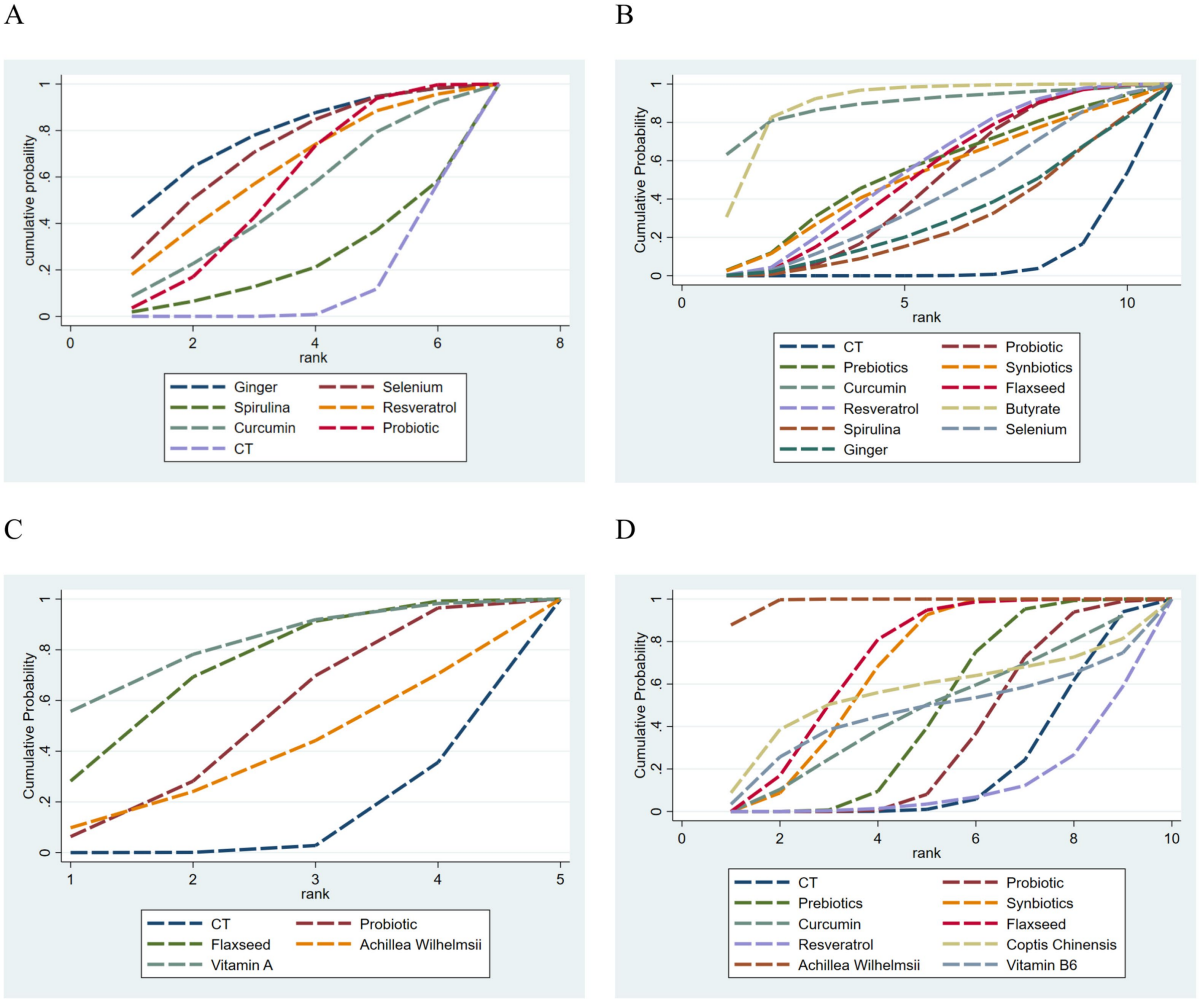
The SUCRA values for principal outcomes. **(A)** Clinical disease activity. **(B)** IBDQ score. **(C)** Mayo score. **(D)** CRP. For the *x*-axis representing rank, rank 1 denotes the best performing intervention.

Based on the ranking of SUCRA values, ginger supplementation appeared to be the most effective intervention for reducing the clinical activity index (80.0%), followed by selenium supplementation (72.9%). However, considering that SUCRA is an indirect comparison, ginger supplements may be the best intervention, but considering the lack of statistical significance between more groups, the ranking results are only used as an exploratory reference. The SUCRA values for the remaining interventions are presented in Figure 5A.

### IBDQ

A total of 12 studies (18, 20, 22–27, 34–36, 40) reported the effects of dietary supplements on Inflammatory Bowel Disease Questionnaire (IBDQ) score improvement in patients with UC, comprising 806 patients and involving 11 interventions. The network geometry of the included interventions is presented in Figure 4B. As the inconsistency model test yielded a *p*-value >0.05, a consistency model was employed for the NMA. The NMA revealed that, in terms of IBDQ score, among 10 dietary supplements compared with conventional drug therapy or placebo, probiotics (MD = 7.78, 95% CI: 1.39 to 14.17), flaxseed extract (MD = 8.58, 95% CI: 2.31 to 14.85), resveratrol (MD = 9.18, 95% CI: 2.45 to 15.91) and butyrate (MD = 20.50, 95% CI: 9.80 to 31.20) demonstrated significant superiority, with statistically significant differences (*p* < 0.05). Curcumin (MD = 25.80, 95% CI: 2.00 to 49.60) also yielded a statistically significant improvement; nevertheless, the exceptionally wide confidence interval indicates high uncertainty, suggesting this should be viewed clearly as a preliminary finding. In intergroup comparisons, butyrate showed significant advantages over spirulina (MD = 16.70, 95% CI: 2.48 to 30.92), ginger supplementation (MD = 16.24, 95% CI: 1.03 to 31.45), and probiotics (MD = 12.72, 95% CI: 0.26 to 25.18), all with statistically significant differences (*p* < 0.05). No statistically significant differences were observed in the other pairwise comparisons among the remaining interventions (*p* > 0.05). The results of all pairwise comparisons are presented in Supplementary Table S3.

Based on the ranking of SUCRA values, butyrate appeared to be the most effective intervention for reducing IBDQ scores (89.9%), followed by curcumin, which also demonstrated a notable effect (89.2%). However, considering the lack of statistical significance between more groups, the ranking results are only used as an exploratory reference. The SUCRA values for the remaining interventions are presented in Figure 5B.

### Mayo score

A total of seven studies (24, 28, 30, 32, 36, 37, 39) reported the effects of dietary supplements on Mayo score improvement in patients with UC, comprising 446 patients and involving five interventions. The network geometry of the included interventions is presented in Figure 4C. As the inconsistency model test yielded a *p*-value >0.05, a consistency model was employed for NMA. The NMA revealed that, in terms of Mayo score, among four dietary supplements compared with conventional drug therapy or placebo, flaxseed extract (MD = −1.53, 95% CI: −2.82 to −0.24) demonstrated significant superiority, with statistically significant differences (*p* < 0.05). Vitamin A (MD = −1.93, 95% CI: −3.82 to −0.04) showed a borderline significant improvement; given the narrow margin, this result warrants a conservative interpretation. No statistically significant differences were observed in the other pairwise comparisons among the remaining interventions (*p* > 0.05). The results of all pairwise comparisons are presented in Supplementary Table S4.

Based on the ranking of SUCRA values, vitamin A appeared to be the most effective intervention for reducing the Mayo score (81.1%), followed by flaxseed extract (72.0%). Vitamin A may be the optimal intervention measure, but considering the lack of statistical significance among the more groups, the ranking result is only an exploratory reference. The SUCRA values for the remaining interventions are presented in Figure 5C.

### C-reactive protein (CRP)

A total of eight studies (17–19, 25, 28, 31, 32, 36) reported the effects of dietary supplements on CRP levels in patients with UC, comprising 384 patients and involving 10 interventions. The network geometry of the included interventions is presented in Figure 4D. As the inconsistency model test yielded a *p*-value >0.05, a consistency model was employed for the NMA. The NMA revealed that, in terms of CRP levels, among nine dietary supplements compared with conventional drug therapy or placebo, synbiotics (MD = −0.69, 95% CI: −1.16 to −0.23), flaxseed extract (MD = −0.80, 95% CI: −1.16 to −0.44), and *Achillea wilhelmsii* (MD = −2.95, 95% CI: −3.52 to −2.38) demonstrated significant superiority, with statistically significant differences (*p* < 0.05). Furthermore, *Achillea wilhelmsii* showed significant advantages over resveratrol, probiotics, prebiotics, curcumin, synbiotics, and flaxseed extract (*p* < 0.05). Flaxseed extract was significantly more effective than resveratrol and probiotics (*p* < 0.05). Synbiotics demonstrated significant superiority over resveratrol, probiotics, and prebiotics (*p* < 0.05). No statistically significant differences were observed in the other pairwise comparisons among the remaining interventions (*p* > 0.05). The results of all pairwise comparisons are presented in Supplementary Table S5.

Based on the ranking of SUCRA values, *Achillea wilhelmsii* appeared to be the most effective intervention for improving CRP levels in patients with UC (98.6%), followed by flaxseed extract (71.3%). However, considering the lack of statistical significance between more groups, the ranking results are only used as an exploratory reference. The SUCRA values for the remaining interventions are presented in Figure 5D.

#### Safety and adverse events

To systematically evaluate the safety profile of the included dietary supplements, we attempted to extract adverse event data from all included trials. However, we found that the vast majority of the original studies did not report any relevant safety data. The limited available data regarding adverse events and safety outcomes have been collated and are presented in Supplementary Table S1. Based on this restricted pool of evidence, the reported adverse events appeared to be generally mild, but the widespread lack of systematic safety reporting in the primary literature prevents a comprehensive safety analysis.

#### Sensitivity analysis

A sensitivity analysis was performed to evaluate the robustness of our findings by excluding the study by Ou et al. (23) (which used an active drug comparator) and the study by Morshedzadeh et al. (36) (which used an undefined conventional treatment). After excluding these studies, the network meta analysis was reanalyzed. The results demonstrated that the primary findings remained stable. For instance, butyrate still showed significant superiority over placebo in improving IBDQ scores (MD = 44.70, 95% CI: 1.63 to 1222.22), confirming that the inclusion of these variations in the control node did not critically distort the main effect estimates. The network plots for IBDQ, Mayo score, and CRP are provided in the Supplementary Figures S1–S3.

### Assessment of publication bias

**Figure 6:**
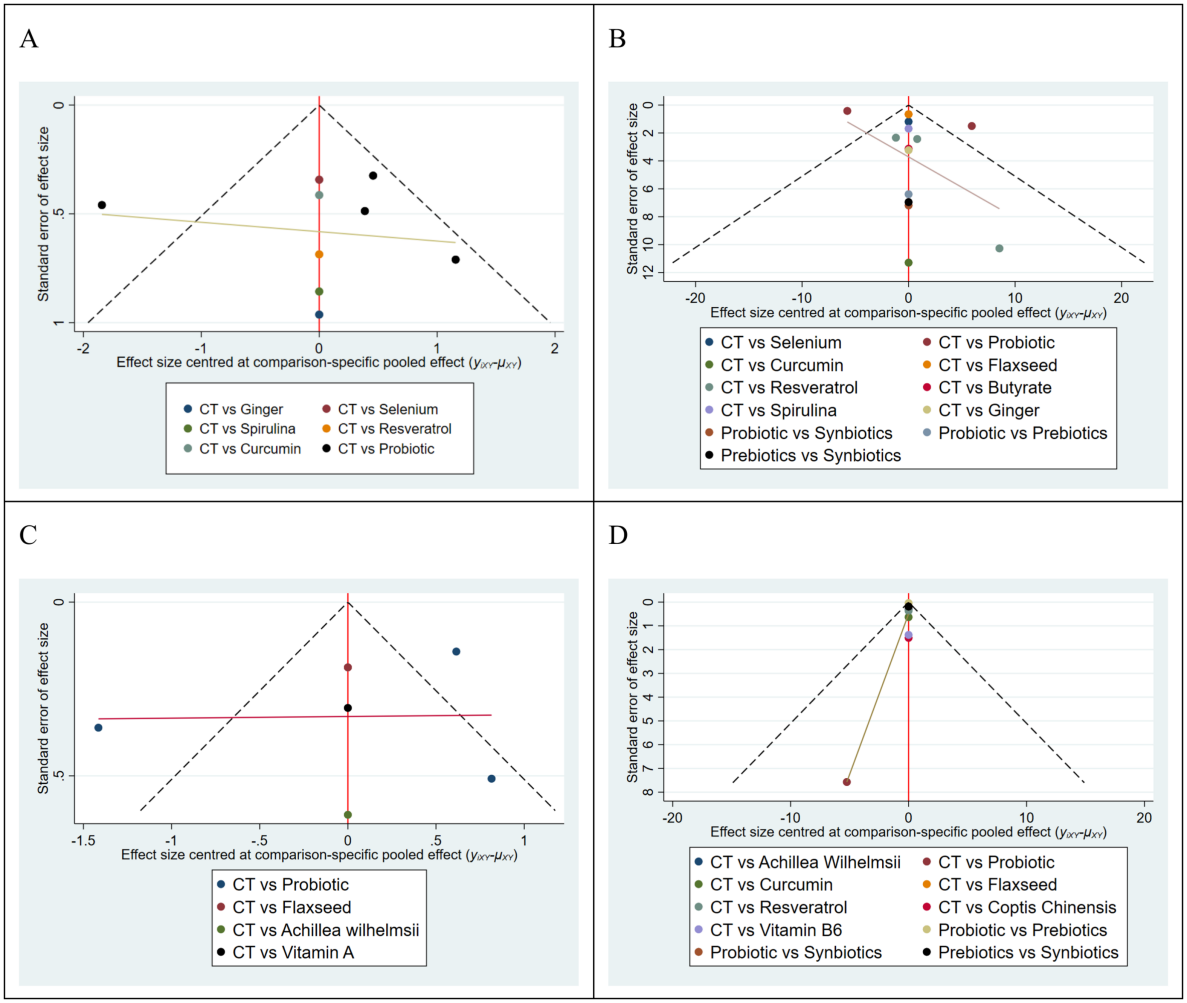
Comparison adjusted funnel plot of each outcome indicator. **(A)** Clinical disease activity. **(B)** IBDQ score. **(C)** Mayo score. **(D)** CRP.

A comparison-adjusted funnel plot was generated to assess small-study effects in the network meta-analysis involving the 24 included studies. Each data point representing an individual study. Visual inspection revealed that the majority of studies were symmetrically distributed around the zero line and fell within the expected funnel boundaries, suggesting a low probability of publication bias. However, some asymmetry was observed, along with a few outliers beyond the funnel limits. These findings may indicate a potential small-study effect, which could be partly attributable to the relatively limited number of studies available for certain comparisons in this network (Figure 6).

### Conventional meta-analysis

Given that a NMA could not be performed for the studies involving two of the outcome measures, a conventional meta-analysis was conducted to evaluate the effects of dietary supplements on erythrocyte sedimentation rate (ESR) and fecal calprotectin (FCAL).

Regarding ESR, a total of seven studies (17, 19, 24, 25, 27, 28, 31) reported the effects of dietary supplements on ESR levels in patients with UC, involving eight interventions and 401 patients. Given the considerable heterogeneity among the included interventions (*I*^2^ = 68.8%, *p* < 0.01), a random-effects model was employed for the analysis. The results of the conventional meta-analysis showed that, compared with the control group, dietary supplements significantly reduced ESR levels in patients with UC (MD = −3.82, 95% CI: −6.02 to −1.61, *p* < 0.01), with a statistically significant difference. Subgroup analysis revealed that, compared with placebo, flaxseed extract (MD = −6.88, 95% CI: −7.99 to −5.77, *p* < 0.01), *Coptis chinensis* extract (MD = −3.10, 95% CI: −5.77 to −0.43, *p* = 0.02), and *Achillea wilhelmsii* (MD = −3.50, 95% CI: −5.78 to −1.22, *p* < 0.01) demonstrated significant advantages, all with statistically significant differences. Due to the considerable heterogeneity observed among the included studies, a sensitivity analysis using the leave-one-out method was conducted to explore the potential sources of heterogeneity. After sequentially excluding the studies by Moradi and Bjarnason, the heterogeneity decreased markedly, suggesting that differences in intervention types may be the primary source of heterogeneity. Details are provided in Supplementary Table S6.

Regarding fecal calprotectin (FCAL), a total of five studies (17, 24, 26, 31, 37) reported the effects of dietary supplements on FCAL levels in patients with ulcerative colitis, involving five interventions and 264 patients. Given the considerable heterogeneity among the included interventions (*I*^2^ = 62.3%, *p* = 0.047), a random-effects model was employed for the analysis. The results of the conventional meta-analysis showed that, compared with the control group, dietary supplements significantly reduced fecal calprotectin (FCAL) levels in patients with ulcerative colitis (MD = −112.45, 95% CI: −201.43 to −23.46, *p* < 0.01), with a statistically significant difference. Subgroup analysis revealed that, compared with placebo, butyrate (MD = −185.40, 95% CI: −269.77 to −101.03, *p* < 0.01) and flaxseed extract (MD = −160.17, 95% CI: −220.26 to −100.08, *p* < 0.01) demonstrated significant advantages, both with statistically significant differences. One study directly compared probiotics with prebiotics, and the results showed that probiotics were significantly superior to prebiotics in reducing FCAL levels (MD = −503.00, 95% CI: −876.92 to −129.08, *p* < 0.01). Due to the considerable heterogeneity among the included studies, a sensitivity analysis using the leave-one-out method was conducted to explore the potential sources of heterogeneity. After excluding the study by Alavinejad, the heterogeneity decreased markedly, suggesting that differences in intervention types may be the primary source of heterogeneity. Details are provided in Supplementary Table S7.

## Discussion

### Summary of the main results

This meta-analysis comprehensively evaluated the comparative efficacy of multiple dietary supplements in improving various clinical and laboratory outcomes in patients with UC. A total of 24 RCTs published between 2004 and 2025, involving 1,491 participants and 14 dietary supplements, were included. Through NMA and conventional meta-analysis, we found that the therapeutic effects of different dietary supplements on UC were intervention-specific, with no single supplement demonstrating overall superiority across all outcomes: in terms of clinical activity index, probiotics and selenium showed significant effects, and ginger supplementation ranked high in SUCRA values, although this finding should be interpreted with caution; for Mayo score, flaxseed extract and vitamin A demonstrated significant advantages; regarding IBDQ score, five supplements including probiotics and flaxseed extract exhibited prominent effects, with butyrate being significantly superior to several other supplements and ranking high in SUCRA; for CRP levels, synbiotics, flaxseed extract, and *Achillea wilhelmsii* showed clear efficacy, with the latter demonstrating advantages over multiple other supplements; conventional meta-analysis revealed that dietary supplements significantly reduced ESR and FCAL levels in UC patients, specifically, flaxseed extract and *Coptis chinensis* extract were effective in lowering ESR, while butyrate and flaxseed extract significantly reduced FCAL, and probiotics were superior to prebiotics in reducing FCAL. In conclusion, dietary supplements demonstrate strong potential in alleviating symptoms and inflammation in patients with UC. However, further studies are warranted to validate their safety and reproducibility.

### Comparison with other studies

In recent years, dietary supplements have garnered widespread attention as a potential adjunctive therapeutic strategy for UC. Previous meta-analyses have evaluated the efficacy of dietary supplements in the treatment of UC. First, the use of vitamin D supplementation in patients with UC has received considerable attention. A systematic review and meta-analysis indicated that vitamin D supplementation significantly improved serum vitamin D levels, erythrocyte sedimentation rate ESR, and CRP levels in UC patients. However, due to the methodological limitations of the included studies, these findings warrant further validation through high-quality studies (41). Second, the combined use of prebiotics and probiotics—referred to as synbiotics—has also been considered beneficial for UC. A systematic review indicated that synbiotics significantly improved colonoscopic and histological scores, clinical activity index, and serum CRP levels in patients. Synbiotics exert their therapeutic effects by increasing beneficial gut microbiota, reducing pro-inflammatory cytokines, and elevating anti-inflammatory cytokine levels. This finding provides strong support for the use of synbiotics as an adjunctive or alternative therapy for UC (11). However, the aforementioned studies were all conventional meta-analyses, which could only compare the efficacy of a single category of dietary supplements in improving UC. As a novel meta-analytic approach, NMA allows for the integration of both direct and indirect comparisons. In the present study, we conducted a comprehensive search of currently available dietary supplements and evaluated their comparative efficacy in the treatment of UC using NMA, aiming to provide a comprehensive assessment.

### Explanation of the research results

In recent years, probiotics have garnered considerable attention as a potential therapeutic strategy due to their role in modulating the gut microbiota and improving intestinal barrier function (42, 43). Studies have shown that probiotics can inhibit the growth of harmful bacteria by enhancing intestinal mucosal barrier function, modulating immune system activity, and promoting the secretion of anti-inflammatory factors. In the treatment of UC, probiotics exert their effects through multiple mechanisms. First, probiotics can restore gut microbial homeostasis by modulating the composition and diversity of the gut microbiota, thereby attenuating inflammatory responses (44). For example, *Lactobacillus plantarum* has been shown to improve intestinal barrier function by upregulating the expression of tight junction proteins, promoting mucin production, and increasing levels of short-chain fatty acids (SCFAs) (45). Furthermore, probiotics play a key role in the immunomodulation of UC by regulating the activity of immune cells, promoting the proliferation of regulatory T cells (Tregs), and inhibiting the activation of pro-inflammatory cells (46, 47). The application of probiotics in UC is not limited to the use of single strains; the use of multi-strain probiotics has also demonstrated favorable therapeutic effects. For example, studies have shown that *Bifidobacterium longum* and its derived extracellular vesicles can alleviate UC by improving the intestinal barrier, regulating immune cell differentiation, and promoting the production of SCFAs (48). In addition, the combination of probiotics with conventional medications has demonstrated a synergistic effect, significantly improving the clinical remission rate (49).

Selenium, as an essential trace element, has demonstrated significant potential in the prevention and treatment of UC. Studies have shown that selenium exerts protective effects against UC through multiple mechanisms, including the inhibition of ferroptosis in intestinal epithelial cells and the enhancement of antioxidant capacity (50, 51), selenium alleviates symptoms of UC by upregulating the expression of glutathione peroxidase 4 (GPX4), thereby inhibiting ferroptosis in intestinal epithelial cells. Studies have found that serum selenium levels in patients with UC are significantly lower than those in healthy individuals and are negatively correlated with disease activity. Furthermore, selenium further enhances its antioxidant and anti-inflammatory effects by regulating the expression of nuclear factor erythroid 2-related factor 2 (Nrf2) and GPX4 (50). In addition to its direct antioxidant effects, selenium exerts its protective role by modulating the gut microbiota and enhancing intestinal barrier function. Selenium-enriched tea polysaccharides can maintain colonic mucosal barrier integrity by upregulating the expression of tight junction proteins, and improve the composition of the gut microbiota by promoting the proliferation of beneficial bacteria and inhibiting the growth of pathogenic bacteria (52). Similarly, selenium-enriched *Bifidobacterium* has shown potential in alleviating UC symptoms by enhancing the intestinal barrier and modulating the gut microbiota (53).

Plant extracts have garnered widespread attention due to their potential anti-inflammatory and antioxidant properties, and have become a research hotspot for alleviating symptoms of UC. Multiple studies have demonstrated that plant extracts exert their effects through various mechanisms, including regulating oxidative stress, inhibiting inflammatory signaling pathways, and improving the gut microbiota. Flaxseed extract has demonstrated significant mucosal protective effects in mouse models of UC. Studies have shown that flaxseed extract attenuates the severity of UC by modulating cytokine levels, enhancing antioxidant activity, and reducing neutrophil infiltration. These effects may be associated with its ability to decrease levels of interferon-γ (IFN-γ) and tumor necrosis factor-α (TNF-α), while increasing interleukin-17 (IL-17) levels (54). *Scutellaria baicalensis* Georgi, a traditional Chinese herbal medicine, has garnered widespread attention due to its potential in alleviating symptoms of UC. Studies have shown that *Scutellaria baicalensis* and its active components exert anti-inflammatory effects through multiple mechanisms, thereby ameliorating the condition of patients with UC. The polysaccharide fraction of Scutellaria baicalensi has been demonstrated to alleviate UC symptoms by improving intestinal barrier function and modulating the gut microbiota. Specifically, the polysaccharide SP2-1 derived from Scutellaria baicalensi has been found to restore intestinal barrier function by upregulating the expression of tight junction proteins, including ZO-1, occludin, and claudin-5, and to significantly increase the levels of SCFAs such as acetate, propionate, and butyrate, thereby ameliorating pathological damage in UC mice (55). Furthermore, the *Scutellaria baicalensis* extract PF2405 has been shown to significantly reduce the histopathological severity in UC mice by inhibiting TNF-α-induced COX-2 expression, highlighting its therapeutic potential in the treatment of UC (56). Furthermore, other medicinal plants, such as *Rhodiola rosea* and *Coix lacryma-jobi* (Job’s tears), have also been found to exert beneficial effects by improving gut microbiota composition and alleviating UC symptoms. Studies have shown that *Rhodiola rosea* and *Coix lacryma-jobi* can promote the growth of beneficial bacteria such as *Lactobacillus* and *Bifidobacterium*, while inhibiting the growth of certain pathogenic bacteria, thereby improving the clinical manifestations of UC (57). In summary, plant extracts exhibit promising potential for improving UC. Through various mechanisms, these natural compounds not only attenuate inflammatory responses and oxidative stress, but also modulate the gut microbiota, thereby enhancing the quality of life of patients with UC.

Butyrate, a SCFA, plays a critical role in the pathogenesis and progression of UC. Studies have shown that butyrate can alleviate UC symptoms and improve intestinal health through multiple mechanisms. First, the anti-inflammatory effects of butyrate in UC have been extensively studied. Butyrate attenuates inflammatory responses by inhibiting the NF-κB signaling pathway and promoting histone deacetylation, thereby demonstrating significant protective effects in experimental colitis models (58). Furthermore, butyrate alleviates inflammation in UC by modulating the Treg/Th17 balance and restoring immune homeostasis (59). These mechanisms highlight the potential therapeutic value of butyrate in the treatment of UC. Secondly, butyrate has also demonstrated significant efficacy in improving intestinal barrier function. Studies have shown that butyrate exerts protective effects in experimental colitis by improving intestinal barrier integrity and inhibiting ferroptosis through the Nrf2/GPX4 signaling pathway (60). Furthermore, butyrate improves intestinal health by promoting autophagy and modulating the gut microbiota (61). These findings indicate that butyrate plays a crucial role in maintaining intestinal barrier function and regulating the gut microecology. However, the metabolism of butyrate in patients with UC may be affected by inflammation. Studies have shown that butyrate oxidation capacity is significantly reduced in UC patients, which may be associated with downregulated expression of genes involved in butyrate transport and oxidation pathways (62). Furthermore, inflammation may reduce the capacity of epithelial cells to utilize butyrate, thereby compromising its therapeutic efficacy (63). Therefore, in the treatment of UC, the impact of inflammation on butyrate metabolism should be taken into consideration.

Furthermore, while SUCRA rankings provide a useful hierarchy of potential efficacy, they must be interpreted cautiously. SUCRA values do not account for the overall quality of the evidence, the width of the confidence intervals, or the number of included studies. For instance, interventions such as curcumin and ginger ranked highly in our analysis, but their estimates were derived from single or small trials with exceptionally wide confidence intervals. Therefore, these high SUCRA rankings indicate potential therapeutic promise rather than definitive clinical superiority. Our conclusions should be viewed in light of this uncertainty, highlighting the need for future large scale randomized controlled trials to confirm these preliminary rankings.

### Limitations of the study

Nevertheless, this study has several limitations. First, the included original studies exhibited heterogeneity in terms of sample size, intervention dosage and duration, patient disease activity, and background treatment regimens, leading to considerable heterogeneity in some analyses (e.g., ESR and FCAL). Although sensitivity analyses were performed to explore the sources of heterogeneity, this may still affect the precision of the results. Second, some findings that demonstrated statistical significance, such as selenium for clinical activity index, vitamin A for Mayo score, and curcumin for IBDQ score, exhibited marginal benefits or exceptionally wide confidence intervals. Because these specific estimates heavily rely on single or small trials, they should be viewed as preliminary. Similarly, interventions like ginger ranked highly in SUCRA values but lacked statistical significance in direct comparisons against placebo; thus, all these findings must be interpreted with caution. Third, there was methodological heterogeneity within the control group node. While most trials were placebo controlled, a few studies used active comparators or conventional treatments without a strict placebo, and some trials, such as Fujimori et al., were multi arm studies without a placebo anchor. Although our sensitivity analyses confirmed the stability of the main results, the lack of a consistent baseline could introduce bias into indirect estimates. Fourth, incomplete outcome data in some included trials might introduce attrition bias. For instance, in the trial by Fujimori et al., only 32 out of 94 randomized patients provided final C reactive protein measurements. Because this trial is one of only eight studies contributing to this specific network, such attrition could potentially bias the pooled effect estimates. Fifth, most studies had short follow-up periods, lacking assessments of long-term efficacy maintenance, safety, and ability to prevent relapse.

## Conclusion

In conclusion, this meta-analysis suggests that dietary supplements may play an adjunctive role in the management of UC, although their efficacy is not universally applicable. These supplements exhibit differential effects across various clinical and laboratory outcomes in UC. Flaxseed extract demonstrated consistent benefits across multiple inflammatory and symptomatic indicators. Butyrate showed particularly strong evidence in improving quality of life and local intestinal inflammation. Meanwhile, probiotics, curcumin, and others also displayed value in specific domains. These findings provide evidence-based support for the use of dietary supplements as adjunctive therapies in UC. When faced with a wide array of dietary supplements, clinicians and patients should make cautious choices based on the best available evidence and individual circumstances, recognizing their role as “complementary” rather than “alternative” to conventional treatment. Future research is warranted to further validate these findings and advance the field toward precision nutrition and supplement-based therapies for UC.

## Data Availability

The original contributions presented in the study are included in the article/Supplementary material, further inquiries can be directed to the corresponding author.
