# Supplementary material for: Efficacy and safety of dietary supplements for the treatment of ulcerative colitis, a network meta-analysis
Source: Front Med (Lausanne). 2026 Apr 22;13:1816535. doi: 10.3389/fmed.2026.1816535 (PMC13143612; doi:10.3389/fmed.2026.1816535)
Supplement: Supplementary file 1 [file Data_Sheet_1.pdf]

Table S. 1 Characteristics of the included studies.

Author (year)	Sample size(n)		Intervention time		Intervention characteristics		Outcomes	Safety and Adverse Events	
	T	C	T	C	T	C		T	C
Bjarnason et al. (2019)	40	41	4 weeks	4 weeks	Probiotic	Placebo	CAI/CRP/FACL/ESR		
Fujimori et al. (2009)[a]	31	31	4 weeks	4 weeks	Probiotic	Prebiotics	IBDQ		
	10[1]	10[1]	4 weeks	4 weeks	Probiotic	Prebiotics	CRP		
Fujimori et al. (2009)[b]	31	32	4 weeks	4 weeks	Probiotic	Synbiotics	IBDQ		
	10[1]	12[1]	4 weeks	4 weeks	Probiotic	Synbiotics	CRP		
Fujimori et al. (2009)[c]	31	32	4 weeks	4 weeks	Prebiotics	Synbiotics	IBDQ		
	10[1]	12[1]	4 weeks	4 weeks	Prebiotics	Synbiotics	CRP		
Malekpour et al. (2025)	28	28	2 months	2 months	Coptis Chin-ensis	Placebo	CRP/ESR		
Samsami-Kor et al. (2015)	25	25	6 weeks	6 weeks	Resveratrol	Placebo	IBDQ		
Hanai et al. (2006)	43	39	6 months	6 months	Curcumin	Placebo	CAI		
Rayyan et al. (2023)	12	12	6 weeks	6 weeks	Probiotic	Placebo	IBDQ		
Ou et al. (2021)	69	73	12 weeks	12 weeks	Probiotic	Mesalamine	IBDQ		
Morshedzadeh et al. (2019)	50	25	12 weeks	12 weeks	Flaxseed ex-tract	Placebo	IBDQ/Mayo Score/FCAL/ESR		
Doğan et al. (2024)[d]	15	16	8 weeks	8 weeks	Curcumin	Placebo	IBDQ/CRP/ ESR		
Doğan et al. (2024)[e]	15	16	8 weeks	8 weeks	Resveratrol	Placebo	IBDQ/CRP/ ESR		
Firoozi et al. (2024)	18	18	12 weeks	12 weeks	Butyrate	Placebo	IBDQ/FCAL		
Moradi et al. (2024)	36	37	8 weeks	8 weeks	Spirulina	Placebo	IBDQ/CAI/ESR		

Author (year)	Sample size(n)		Intervention time		Intervention characteristics		Outcomes	Safety and Adverse Events	
	T	C	T	C	T	C		T	C
Amiri et al. (2019)	20	20	1 month	1 month	Achillea wil-helmsii	Placebo	Mayo Score/CRP/ESR	skin rash (contact dermatitis)	
Ikegami et al. (2023)	18	19	8 weeks	8 weeks	Probiotic	Placebo	CAI	diarrhoea and bloody stools	diarrhoea and bloody stools
Tamaki et al. (2016)	24	23	8 weeks	8 weeks	Probiotic	Placebo	Mayo Score	dry cough	
Alavinejad et al. (2020)	15	15	6 months	6 months	Vitamin B6	Placebo	FCAL/CRP/ ESR		
Agraib et al. (2022)	12	12	6 weeks	6 weeks	Probiotic	Placebo	Mayo Score/CRP		
Kato et al. (2004)	10	9	12 weeks	12 weeks	Probiotic	Placebo	CAI		
Samsamikor et al. (2016)	28	28	6 weeks	6 weeks	Resveratrol	Placebo	IBDQ/CAI		
Khazdouz et al. (2023)	44	45	10 weeks	10 weeks	Selenium	Placebo	IBDQ/CAI		
Morshedzadeh et al. (2021)	50	25	12 weeks	12 weeks	Flaxseed ex-tract	Conventi on-al treatme-nt	IBDQ/Mayo Score/CRP		
Tamizifar et al. (2023)	18	24	16 weeks	16 weeks	Probiotic	Placebo	Mayo Score/FCAL		
Sood et al. (2009)	55	29	12 weeks	12 weeks	Probiotic	Placebo	CAI	abdominal bloating and discomfort	
Shirazi et al. (2018)	72	71	2 months	2 months	Vitamin A	Placebo	Mayo Score		
Nikkhah-Bodaghi et al. (2019)	22	24	12 weeks	12 weeks	Ginger extract	Placebo	IBDQ/CAI		

[1]For CRPs, only 32 out of the 94 randomized participants provided final data; T: Treatment group; C: Control group; CAI: clinical activity index; CRP: C-Reactive Protein; ESR: Erythrocyte Sedimentation Rate; FCAL: Fecal Calprotectin; IBDQ: Inflammatory Bowel Disease Quality of Life score.

Table S. 2 Relative effects of different dietary patterns on CAI.

Ginger extract						
-0.47 (-4.33,3.39)	Selenium					
-0.91 (-4.94,3.12)	-0.44 (-4.06,3.18)	Resveratrol				
-1.26 (-4.51,2.00)	-0.79 (-3.52,1.95)	-0.34 (-3.31,2.62)	Probiotic			
-1.40 (-5.282.48)	-0.93 (-4.39,2.53)	-0.49 (-4.13,3.15)	-0.14 (-2.92,2.63)	Curcumin		
-2.72 (-6.87,1.43)	-2.25 (-6.01,1.51)	-1.81 (-5.74,2.12)	-1.46 (-4.60,1.67)	-1.32 (-5.10,2.46)	Spirulina	
-2.90 (-5.90,0.10)	-2.43 (-4.85,-0.01)	-1.99 (-4.68,0.70)	-1.64 (-2.91,-0.38)	-1.50 (-3.96,0.96)	-0.18 (-3.05,2.69)	Placebo

Table S. 3 Relative effects of different dietary patterns on IBDQ score.

Butyrate										
-5.30 (-31.40, 20.80)	Curcumin									
11.32 (-1.32,23.96)	16.62 (-8.12,41.35)	Resveratrol								
10.92 (-6.92,28.76)	16.22 (-11.53,43.98)	-0.40 (-16.16, 15.37)	Prebiotics							
11.92 (-0.48,24.32)	17.22 (-7.39,41.84)	0.60 (-8.59,9.80)	1.00 (-14.59,16.59)	Flaxseed extract						
11.52 (-6.71,29.75)	16.82 (-11.19,44.83)	0.20 (-16.00,16.41)	0.60 (-12.56,13.76)	-0.40 (-16.44,15.64)	Synbiotics					
12.72 (0.26,25.18)	18.02 (-6.63,42.67)	1.40 (-7.84,10.65)	1.80 (-10.96,14.56)	0.80 (-8.15,9.75)	1.20 (-12.11,14.51)	Probiotic				
14.01 (-0.02,28.04)	19.31 (-6.16,44.78)	2.69 (-8.60,13.99)	3.09 (-13.82,20.00)	2.09 (-8.93,13.11)	2.49 (-14.84,19.81)	1.29 (-9.81,12.38)	Selenium			
16.24 (1.03,31.45)	21.54 (-4.61,47.69)	4.92 (-7.82,17.66)	5.32 (-12.59,23.23)	4.32 (-8.18,16.82)	4.72 (-13.58,23.02)	3.52 (-9.04,16.08)	2.23 (-11.89,16.35)	Ginger extract		
16.70 (2.48,30.92)	22.00 (-3.58,47.58)	5.38 (-6.15,16.92)	5.78 (-11.30,22.85)	4.78 (-6.49,16.05)	5.18 (-12.31,22.66)	3.98 (-7.36,15.32)	2.69 (-10.35,15.73)	0.46 (-13.85,14.77)	Spirulina	
20.50 (9.80,31.20)	25.80 (2.00,49.60)	9.18 (2.45,15.91)	9.58 (-4.70,23.85)	8.58 (2.31,14.85)	8.98 (-5.78,23.74)	7.78 (1.39,14.17)	6.49 (-2.58,15.56)	4.26 (-6.56,15.08)	3.80 (-5.57,13.17)	Placebo

Table S. 4 Relative effects of different dietary patterns on Mayo score.

Vitamin A				
-0.40 (-2.69,1.89)	Flaxseed extract			
-1.01 (-3.21,1.18)	-0.61 (-2.32,1.09)	Probiotic		
-1.38 (-4.25,1.49)	-0.98 (-3.49,1.53)	-0.37 (-2.79,2.06)	Achillea wilhelmsii	
-1.93 (-3.82,-0.04)	-1.53 (-2.82,-0.24)	-0.92 (-2.03,0.19)	-0.55 (-2.71,1.61)	Placebo

Table S. 5 Relative effects of different dietary patterns on CRP.

Achillea wil-helmsii									
-2.15 (-2.83,-1.47)	Flaxseed extract								
-2.26 (-3.00,-1.52)	-0.11 (-0.70,0.48)	Synbiot-ics							
-2.15 (-5.15,0.85)	-0.00 (-2.97,2.97)	0.11 (-2.88,3.09)	Coptis Chinen-sis						
-2.53 (-3.89,-1.17)	-0.38 (-1.67,0.91)	-0.27 (-1.60,1.05)	-0.38 (-3.58,2.82)	Curcu-min					
-2.62 (-3.31,-1.92)	-0.47 (-1.00,0.07)	-0.36 (-0.62,-0.10)	-0.47 (-3.44,2.51)	-0.09 (-1.39,1.21)	Prebiot-ics				
-2.56 (-5.33,0.21)	-0.41 (-3.14,2.32)	-0.30 (-3.05,2.44)	-0.41 (-4.41,3.59)	-0.03 (-3.01,2.95)	0.06 (-2.68,2.79)	Vitamin B6			
-2.69 (-3.38,-2.00)	-0.54 (-1.07,-0.01)	-0.43 (-0.69,-0.17)	-0.54 (-3.51,2.44)	-0.16 (-1.45,1.14)	-0.07 (-0.15,0.01)	-0.13 (-2.86,2.61)	Probiotic		
-2.95 (-3.52,-2.38)	-0.80 (-1.16,-0.44)	-0.69 (-1.16,-0.23)	-0.80 (-3.75,2.15)	-0.42 (-1.66,0.82)	-0.33 (-0.73,0.06)	-0.39 (-3.10,2.32)	-0.26 (-0.65,0.12)	Placebo	
-3.26 (-4.18,-2.34)	-1.11 (-1.92,-0.30)	-1.00 (-1.86,-0.14)	-1.11 (-4.14,1.92)	-0.73 (-2.16,0.70)	-0.64 (-1.47,0.18)	-0.70 (-3.50,2.10)	-0.57 (-1.39,0.25)	-0.31 (-1.03,0.41)	Resvera-trol

Table S. 6

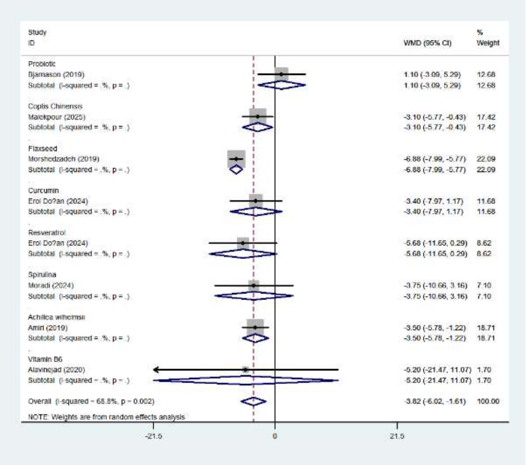

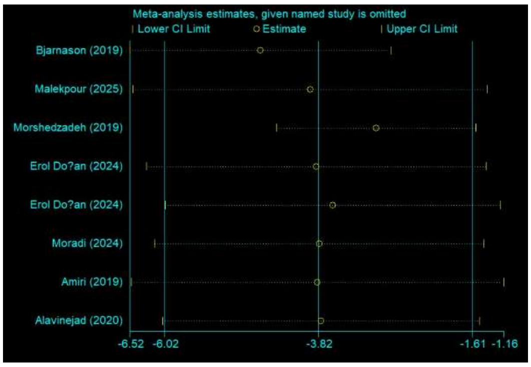

Probiotic	z= 0.51	p = 0.607
Coptis Chinensis	z= 2.27	p = 0.023
Flaxseed extract	z= 12.10	p = 0.000
Curcumin	z= 1.46	p = 0.144
Resveratrol	z= 1.87	p = 0.062
Spirulina	z= 1.06	p = 0.287
Achillea wilhelmsii	z= 3.00	p = 0.003
Vitamin B6	z= 0.63	p = 0.531
Overall	z= 3.39	p = 0.001

Table S. 7

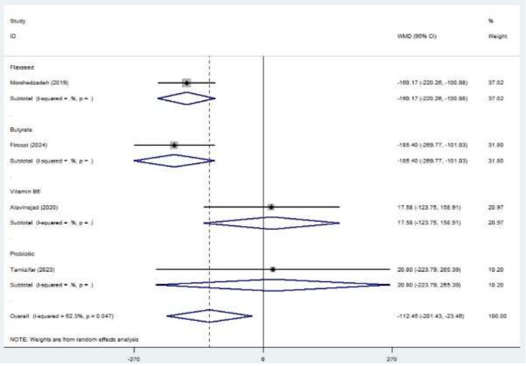

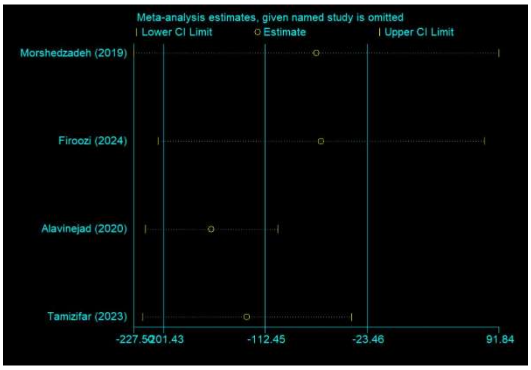

Flaxseed extract	z= 5.22	p = 0.000
Butyrate	z= 4.31	p = 0.000
Vitamin B6	z= 0.24	p = 0.807
Probiotic	z= 0.17	p = 0.868
Overall	z= 2.48	p = 0.013

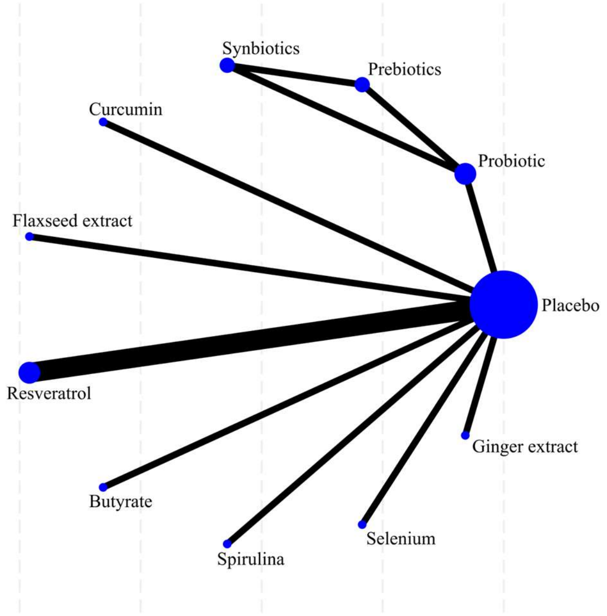

Figure S1. Network plot of interventions for IBDQ score

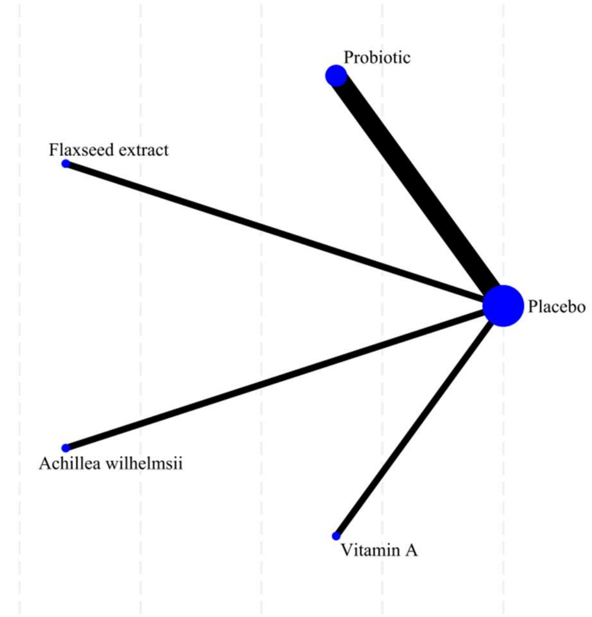

Figure S2. Network plot of interventions for Mayo score

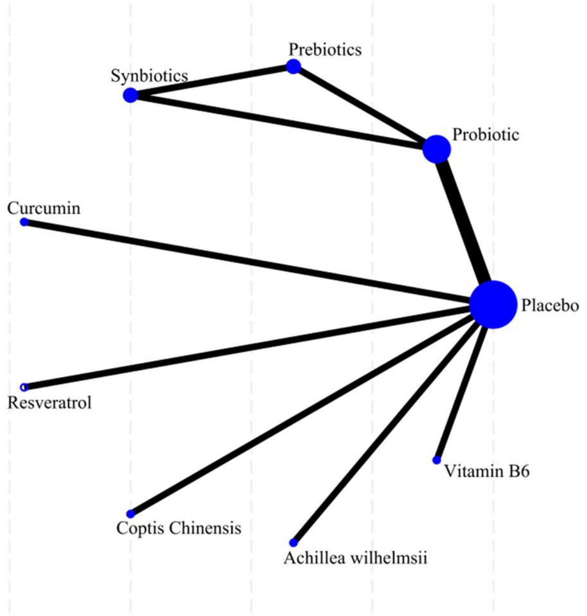

Figure S3. Network plot of interventions for CRP level
